# Adding Sodium–Glucose Co-Transporter 2 Inhibitors to Sulfonylureas and Risk of Hypoglycemia: A Systematic Review and Meta-Analysis of Randomized Controlled Trials

**DOI:** 10.3389/fendo.2021.713192

**Published:** 2021-10-21

**Authors:** Meng Jiang, Qiaoshu Liu, Tiejian Jiang, Paul Nizigiyimana, Minxiang Lei

**Affiliations:** Department of Endocrine, Xiangya Hospital of Central South University, Changsha, China

**Keywords:** SGLT-2 (sodium glucose co transporter 2) inhibitors, sulfonylureas, type 2 diabetes, meta – analysis, systematic review

## Abstract

**Background:**

Hypoglycemia is an important event that could be related to increased mortality in patients with diabetes. The risk of hypoglycemia is not clearly illustrated to increase when Sodiumglucose co-transporter 2 (SGLT-2) inhibitors are used concomitantly with sulfonylureas. The present study will assess the risk of hypoglycemia associated with the concomitant use of SGLT-2 inhibitors and sulfonylureas compared with placebo and sulfonylureas.

**Method:**

We searched Medline, EMBASE, Cochrane Central Register of Controlled Trials, and Clinicaltrial.gov and identified the randomized trials comparing SGLT-2 inhibitors with placebo for type 2 diabetes treated with sulfonylureas. The risk of bias in each trial was assessed using the Cochrane tool. The risk ratio of hypoglycemia was measured using the Mantel Haenszel method. We also performed subgroup analysis to examine the dosage effects. The number needed to harm (NNH) was measured according to the duration of intervention.

**Results:**

A total of 12 studies, including 3761 participants, were enrolled in our systematic review and meta-analysis. The risk ratio of hypoglycemia was 1.67 (95% CI 1.42 to 1.97). The NNH was 13 (95% CI 9 to 21) for a treatment duration of 24 weeks or less, 11 (8 to 18) for 25 to 48 weeks, and 7 (5 to 10) for more than 48 weeks. Subgroup analysis showed that no difference was found between higher and lower doses of SGLT-2 inhibitors. The risk ratio related to lower dose SGLT-2 inhibitors was 1.56 (95% CI 1.30 to 1.88), and the risk ratio related to higher dose SGLT-2 inhibitors was 1.70 (95% CI 1.42 to 2.04).

**Conclusions:**

The risk of hypoglycemia was significantly increased in subjects treated with SGLT-2 inhibitors compared with placebo. Addition of SGLT-2 inhibitors to sulfonylureas would lead to one more case of hypoglycemia in every 13 patients with a treatment duration less than 24 weeks. This suggests that a decrease in sulfonylureas dose may be an important recommendation when adding SGLT-2 inhibitors to sulfonylureas.

## Introduction

Hypoglycemia is a common adverse effect of antihyperglycemic agents. There is a strong relationship between hypoglycemia and increased risk of cardiovascular disease, and mortality. Although glycemic control reduced the cardiovascular events in diabetic patients, intensive glycemic control neither resulted in reduction of cardiovascular events nor decreased all-cause mortality in type 2 diabetic patients (1). Moreover, data from the Action to Control Cardiovascular Risk in Diabetes (ACCORD) trial demonstrated that intensive treatment increased all-cause mortality and cardiovascular mortality compared with standard treatment, which possibly related to the negative effect of hypoglycemia (2).

The hospital admissions rate for hypoglycemia have declined slowly since 2007, while hypoglycemia is still the main complication of old diabetic patients (>60 years) with longer disease duration (3, 4). The immediate effects of hypoglycemia can affect the everyday activities because of impairment of balance, coordination, vision and level of consciousness, and cause vehicle accidents, falls and injury (5, 6). In addition, hypoglycemia induces consequences well beyond its immediate effects. Hypoglycemia is able to frustrate the goal of glycemic control, which limits full archiving of the benefit from improvement in glycemic control (5, 7). It also impairs the awareness of hypoglycemia because of deficient counter regulatory hormonal responses to hypoglycemia (8), which would greatly increase the risk of severe hypoglycemia (8, 9).

SGLT-2 inhibitors are a new class of oral antihyperglycemic drugs. SGLT-2 inhibitors improve hyperglycemia by inhibiting renal glucose reabsorption (10) and decrease cardiovascular events and kidney damage in patients with diabetes (11).

A variety of randomized clinical trials have studied SGLT-2 inhibitors both as monotherapy and in patients treated with other glucose lowering drugs, DPP-4 inhibitors, metformin, sulfonylureas, and thiazolidinediones (12). The risk of hypoglycemia with SGLT-2 inhibitors is similar to that of placebo or other oral agents (metformin, DPP-4 inhibitors, thiazolidinediones, and α-Glucosidase inhibitors) when used as monotherapy (12–16). When used in patients with metformin, all above oral agents associate with the similar HbA1c content, while SGLT-2 inhibitors have the lowest hypoglycemia risk (16). Previous studies reported that SGLT-2 in conjunction with sulfonylureas increased incidence of hypoglycemia significantly (14, 17). This could be related to the higher risk of hypoglycemia among subjects treated with sulfonylureas, which is further increased when patients are treated by a second antidiabetic agent. The summaries of the product characteristics of SGLT-2 inhibitors suggested that the hypoglycemia risk is common due to association with sulfonylureas (18–20). However, some clinical trials reported that SGLT-2 plus sulfonylureas did not increase incidence of hypoglycemia (21, 22). Therefore, this risk is not clearly illustrated yet. We assessed the risk of hypoglycemia associated with the use of SGLT-2 inhibitors and sulfonylureas in patients with type 2 diabetes.

## Methods

### Systematic Review Protocol and Registration

The systematic review and meta-analysis is reported in accordance with the preferred Reporting Items for Systematic Reviews and Meta-Analyses (PRISMA). All research was conducted according to protocol registered in the PROSPERO database (CRD42018096476).

### Search Strategy and Study Selection

We did a search in the PUBMED, EMBASE, and the Cochrane Central Register of Controlled Trials on 10 Apr 2021 using keywords related to SGLT-2 inhibitors and randomized controlled trials (supplement 1). There were no language restrictions. We also screened the reference list of identified studies and performed forward citation searches to consider studies not identified by our online search. In addition, we checked Clinicaltrials.gov (last search April 2021) to identify unpublished but eligible trials.

We identify the eligible trials for this meta-analysis were those that: They were randomized clinical trials in adults with type 2 diabetes; studied the effect of adding one SGLT2 inhibitor to sulfonylureas, with or without other antidiabetic drugs; studied one SGLT2 inhibitor used at daily doses approved in clinical practice-luseogliflozin (Takeda Pharmaceutical), dapagliflozin (Astra-Zeneca), ipragliflozin (Astellas Pharma Inc), canagliflozin (Janssen Research & Development, LLC), empagliflozin (Boehringer Ingelheim), ertugliflozin (Merck Sharp & Dohme Corp), and included no less than 50 patients treated with SGLT2 inhibitors. Studies concerning extension phases of randomized controlled trials were excluded.

Two investigators (MJ and ML) reviewed the titles and abstracts of potentially relevant trials and determined final eligibility through full-text assessment. Disagreements were resolved through discussion with another investigator.

### Data Extraction and Risk of Bias

The following information was collected from each study: number of subjects, sex, age, country, setting, study duration, and change in glycated hemoglobin A1C (HbA1c) level; intervention details (SGLT-2 inhibitors and sulfonylureas names, doses, and number of treated patients); and the number of patients with any hypoglycemia. Disagreements were resolved through discussion with another investigator.

Three categories of hypoglycemia were captured: total hypoglycemia (irrespective of definition, severity, blood glucose value or documentation), severe hypoglycemia (a hypoglycemic event that required the assistance of third party), hypoglycaemia (plasma glucose levels ≤ 3.9mmol/L).

The risk of bias of each study was assessed by using the Cochrane Collaboration risk of bias tool in randomized trials. The quality of the studies was determined by examination of the original study protocol. We graded random sequence generation; allocation concealment; blinding of participants and personnel, blinding of outcome assessment; incomplete outcome data; selective outcome reporting; and other sources of biases. Two investigators independently graded the seven parts as low, unclear or high risk of bias. The quality of evidence resulting from a meta-analysis would be rated from very low to high using the GRADE framework.

### Data Analysis

We compared the risk of hypoglycemia in patients treated with SGLT-2 inhibitors plus sulfonylureas with that in patients treated with placebo plus sulfonylureas.

We calculated the risk ratio for hypoglycemia along with the 95% confidence interval. The fixed effect model (Mantel Haenszel method) was used to calculate the pooled risk ratio.

Statistical heterogeneity was assessed with the I^2^ statistic, considering values below 50% indicative of low heterogeneity (23). The subgroup analysis was undertaken based on the dosage of SGLT-2 inhibitors (**eTable 1** in the supplement).

Publication bias was tested by inspection of funnel plots and Egger’s tests (P<0.05 considered to be significant). The number needed to harm (NNH) (ie, number of patients who receive treatment that would be associated with 1 hypoglycemia) was measured according to the Cochrane recommendations. On the assumption that the risk of hypoglycemia is associated with the duration of treatment, we calculated the assumed control risk for different follow-up scenarios: 24 weeks or less, 25 to 48 weeks, and more than 48 weeks.

We also conducted a secondary analysis according to different doses of SGLT-2 inhibitors. We classified the SGLT-2 inhibitors doses into a lower dose group and higher dose group (**eTable 1** in the supplement) according to previous studies (24) (low daily doses are mostly recommended in patients with renal impairment).

## Results

### Study Selection

**Figure 1:**
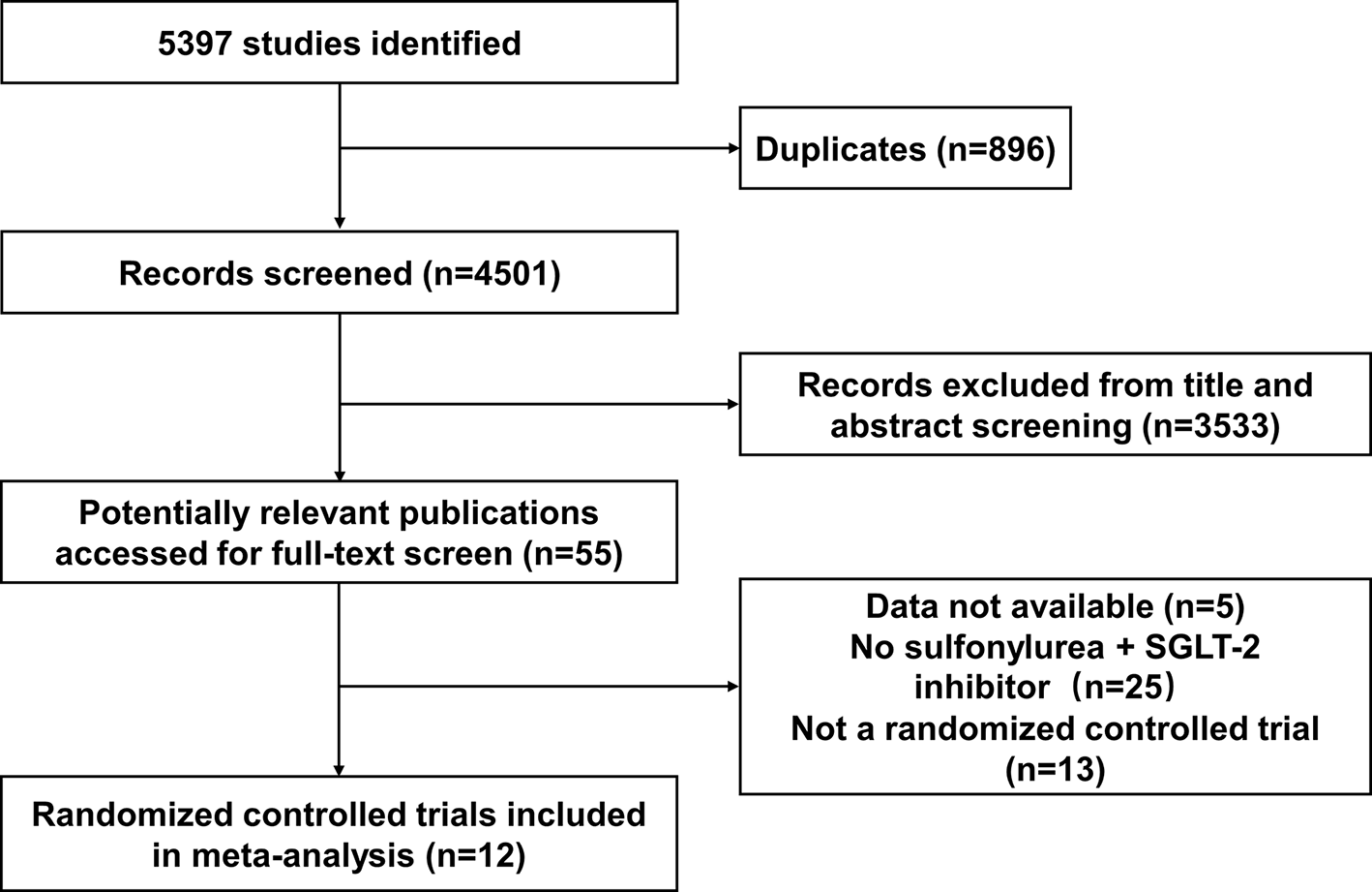
Flow diagram of study identification, selection, and inclusion.

Twelve studies were identified as being eligible for this meta-analysis and systemic review (**Figure 1**). The total number of participants from the 12 studies was 3761, 2529 of whom received SGLT-2 inhibitors plus sulfonylureas and 1232 placebo plus sulfonylureas. One study included only subjects aged 55 years or more. In eight of the 12 studies (17, 21, 22, 25–33) the planned follow-up was 24 weeks or less; one study followed patients for a median time of 48 weeks; three studies followed patients for a median time of over 48 weeks. It was impossible to get specific NNHs for the follow-up period range. Therefore, we calculated the assumed control risk of hypoglycemia in patients treated with sulfonylureas from another meta-analysis (34, 35). The assumed control risk was 11.6% for follow-up of 24 weeks or less (seven studies), 13.3% from 25 to 48 weeks (nine studies), and 22.8% for over 48 weeks (11 studies).

The characteristics of studies included in the present study are summarized in **Table 1**. The type of associated sulfonylureas varied across the trials. Five studies enrolled patients only receiving sulfonylureas, while the remaining trials recruited receiving sulfonylureas and metformin. Baseline patient characteristics (mean glycated hemoglobin A1C, mean age, and sex) were well balanced among the patients in each of the groups. In addition, the severe and all hypoglycemic events are summarized in **Table 2**. The severe hypoglycemic events were reported in six studies.

**Table 1:** Characteristics of included studies.

Reference	Study duration (weeks)	Intervention, daily dose (No of patients)	Associated sulfonylureas	Mean hemoglobin A1c at baseline (%)	Mean age of participants (years)	Male (%)	Definition of hypoglycemia
Seino et al. (21)	24	Luseogliflozin 5 mg (n=150) or placebo (n=71)	Glimepiride	SGLT-2 inhibitors: 8.07, placebo: 8.01	SGLT-2 inhibitors: 61, placebo: 60	SGLT-2 inhibitors: 75, placebo: 68	≤3.9 mmol/l with or without symptoms
Matthaei et al. (16)	24	Dapagliflozin 10 mg (n=108) or, placebo (n=108)	Sulfonylureas, not specified	SGLT-2 inhibitors: 8.08, placebo: 8.24	SGLT-2 inhibitors: 61, placebo: 61	SGLT-2 inhibitors:44, placebo: 56	Capillary or plasma glucose measurement <3.5 mmol/L, with or without symptoms
Kashiwagi et al. (22)	24	Ipragliflozin 50 mg (n=52) or, placebo(n=20)	Sulfonylureas, not specified	SGLT-2 inhibitors: 7.53, placebo: 7.55	SGLT-2 inhibitors: 64, placebo: 66	SGLT-2 inhibitors: 78, placebo: 78.3	NR
Ji et al. (23)	18	Canagliflozin 100 mg (n=115) or 300 mg (n=115) or, placebo (n=116)	Sulfonylureas, not specified	SGLT-2 inhibitors:8.0, placebo: 7.9	SGLT-2 inhibitors: 56-57, placebo: 56,	SGLT-2 inhibitors: 53, placebo: 55	≤3.9 mmol/l with or without symptoms
Haering et al. (24)	24	Empagliflozin 10 mg (n=225) or 25 mg (n=216) or, placebo (n=225)	Sulfonylureas, not specified	SGLT-2 inhibitors: 8.07-8.10, placebo: 8.15	SGLT-2 inhibitors: 57, placebo: 57,	SGLT-2 inhibitors: 51, placebo: 50	≤3.9 mmol/l with or without symptoms
Yale et al. (25)	52	canagliflozin 100 mg (n=74) or 300 mg (n=72) or, placebo (n=69)	Sulfonylureas, not specified	SGLT-2 inhibitors: 8.1-8.3, placebo: 8.4	SGLT-2 inhibitors: 64-66, placebo: 64	SGLT-2 inhibitors: 54, placebo: 59,	≤ 3.9 mmol/l with or without symptoms
Strojek et al. (26)	48	Dapagliflozin 2.5 mg (n=154) or 5mg (n=142) or 10mg (n=151) or, placebo(n=145)	Glimepiride	SGLT-2 inhibitors: 8.07-8.12, placebo: 8.15	SGLT-2 inhibitors: 58.9- 60.2, palcebo: 60,	SGLT-2 inhibitors: 48, placebo: 49,	either a symptomatic episode with a capillary or plasma glucose measurement ≤ 3.5 mmol/L
Wilding et al. (27)	52	Canagliflozin 100 mg (n=157) or 300 mg (n=156) and placebo (n=156)	Sulfonylureas, not specified	SGLT-2 inhibitors: 8.1, placebo: 8.1	SGLT-2 inhibitors: 1-57.4, palcebo:56.8	SGLT-2 inhibitors: 52, placebo:49,	≤ 3.9 mmol/l with or without symptoms
Fulcher et al. (28)	18	Canagliflozin 100 mg (n=42) or 300 mg (n=40) and placebo (n=45)	Sulfonylureas, not specified	SGLT-2 inhibitors: 8.2-8.3, placebo: 8.5	SGLT-2 inhibitors: 64.1-65.5, placebo:64.8	SGLT-2 inhibitors: 56, placebo:58,	≤ 3.9 mmol/l with or without symptoms
Blonde et al. (29)	104	Canagliflozin 100mg (n=121) or 300 mg (n=116) and placebo (n=111)	Sulfonylureas, not specified	SGLT-2 inhibitors: 7.7-7.8, placebo: 7.8	SGLT-2 inhibitors: 63-64, placebo:63	SGLT-2 inhibitors: 53, placebo:60,	≤3.9 mmol/l with or without symptoms
Bufoff et al. (33)	18	Ertugliflozin 5 mg(n=100) or 15 mg(n=113) and placebo (n=117)	Sulfonylureas, not specified	SGLT-2 inhibitors: 8.3-8.4, placebo: 8.3	SGLT-2 inhibitors: 62.7-63.2, placebo:63.7	SGLT-2 inhibitors: 73, placebo:78,	≤3.9 mmol/l with or without symptoms
Stroiek et al. (32)	18	Ertugliflozin 5 mg(n=55) or 15 mg(n=54) and placebo (n=48)	Sulfonylureas, not specified	SGLT-2 inhibitors: 8.3-8.4, placebo: 8.2	SGLT-2 inhibitors: 64.3-64.9, placebo:64.4	SGLT-2 inhibitors: 58, placebo:35,	≤3.9 mmol/l with or without symptoms

SGLT-2, Sodium-glucose Cotransporter-2; hemoglobin A1c, glycated hemoglobin; NR, not reported.

**Table 2:** The hypoglycemia events of included studies.

Reference	Study duration (weeks)	Intervention, daily dose (No of patients)	All hypoglycemia	Severe hypoglycemia
Seino et al. (25)	24	Luseogliflozin 5 mg (n=150) or, placebo (n=71)	SGLT-2 inhibitors: (13/150)	SGLT-2 inhibitors: (0/150)
			Placebo: (3/71)	Placebo: (0/71)
Matthaei et al. (17)	24	Dapagliflozin 10 mg (n=108) or, placebo (n=108)	SGLT-2 inhibitors: (4/109)	SGLT-2 inhibitors: (0/109)
			Placebo: (14/109)	Placebo: (0/109)
Kashiwagi et al. (22)	24	Ipragliflozin 50 mg (n=52) or, placebo(n=20)	SGLT-2 inhibitors: (1/52)	SGLT-2 inhibitors: (0/52)
			Placebo: (0/20)	Placebo: (0/20)
Ji et al. (26)	18	Canagliflozin 100 mg (n=115) or 300 mg (n=115) or, placebo (n=116)	SGLT-2 inhibitors: (39/230)	SGLT-2 inhibitors: (3/230)
			Placebo: (9/116)	Placebo: (0/116)
Haering et al. (27)	24	Empagliflozin 10 mg (n=225) or 25 mg (n=216) or, placebo (n=225)	SGLT-2 inhibitors: (61/441)	SGLT-2 inhibitors: (0/441)
			Placebo: (19/225)	Placebo: (0/225)
Yale et al. (25)	52	canagliflozin 100 mg (n=74) or 300 mg (n=72) or, placebo (n=69)	SGLT-2 inhibitors: (19/146)	SGLT-2 inhibitors: (1/146)
			Placebo: (10/69)	Placebo: (0/69)
Strojek et al. (28)	48	Dapagliflozin 2.5 mg (n=154) or 5mg (n=142) or 10mg (n=151) or, placebo(n=145)	SGLT-2 inhibitors: (47/447)	SGLT-2 inhibitors: (1/447)
			Placebo: (10/145)	Placebo: (0/145)
Wilding et al. (29)	52	Canagliflozin 100 mg (n=157) or 300 mg (n=156) or, placebo (n=156)	SGLT-2 inhibitors: (110/313)	SGLT-2 inhibitors: (2/313)
			Placebo: (28/156)	Placebo: (1/156)
Greg et al. (30)	18	Canagliflozin 100 mg (n=42) or 300 mg (n=40) or, placebo (n=45)	SGLT-2 inhibitors: (6/82)	SGLT-2 inhibitors: (0/82)
			Placebo: (2/45)	Placebo: (0/45)
Blonde et al. (31)	104	Canagliflozin 100mg (n=121) or 300 mg (n=116) or, placebo (n=111)	SGLT-2 inhibitors: (125/237)	SGLT-2 inhibitors: (3/237)
			Placebo: (40/111)	Placebo: (4/111)
Bufoff et al. (33)	18	Ertugliflozin 5 mg(n=100) or 15 mg(n=113) or, placebo (n=117)	SGLT-2 inhibitors: (50/213)	SGLT-2 inhibitors: (4/213)
			Placebo: (17/117)	Placebo: (1/117)
Stroiek et al. (32)	18	Ertugliflozin 5 mg(n=55) or 15 mg(n=54) or, placebo (n=48)	SGLT-2 inhibitors: (9/109)	SGLT-2 inhibitors: (0/109)
			Placebo: (2/48)	Placebo: (0/48)

SGLT-2, Sodium-glucose Cotransporter-2.

One trial studied luseogliflozin 2.5 mg/day in a total of 150 patients (25). Dapagliflozin 2.5 mg/day(n=154) was studied in one trial (28) and dapagliflozin 5 mg/day (n=142) in one trial (28) and dapagliflozin 10 mg/day (n=170) in two trials (17, 28). Ipragliflozin was studied at 50 mg/day (n=52) in one trial (22). Canagliflozin was studied at 100 mg/day (n=509) to 300 mg/day (n=499) in 5 trials (21, 26, 29–31). Empagliflozin (10 mg/day (n=225) and 25 mg/day (n=216)) was studied in one trial (27). Ertugliflozin (5 mg/day (n=155) and 10 mg/day (n=167)) was studied in two trials. Overall, 1232 patients receiving placebo plus sulfonylureas were identified in the 12 included trials (**Table 1**). One of the 12 trials failed to clearly report the definition of hypoglycemia (**Table 1**) (22, 25).

### Risk of Bias Assessment

**Figure 2:**
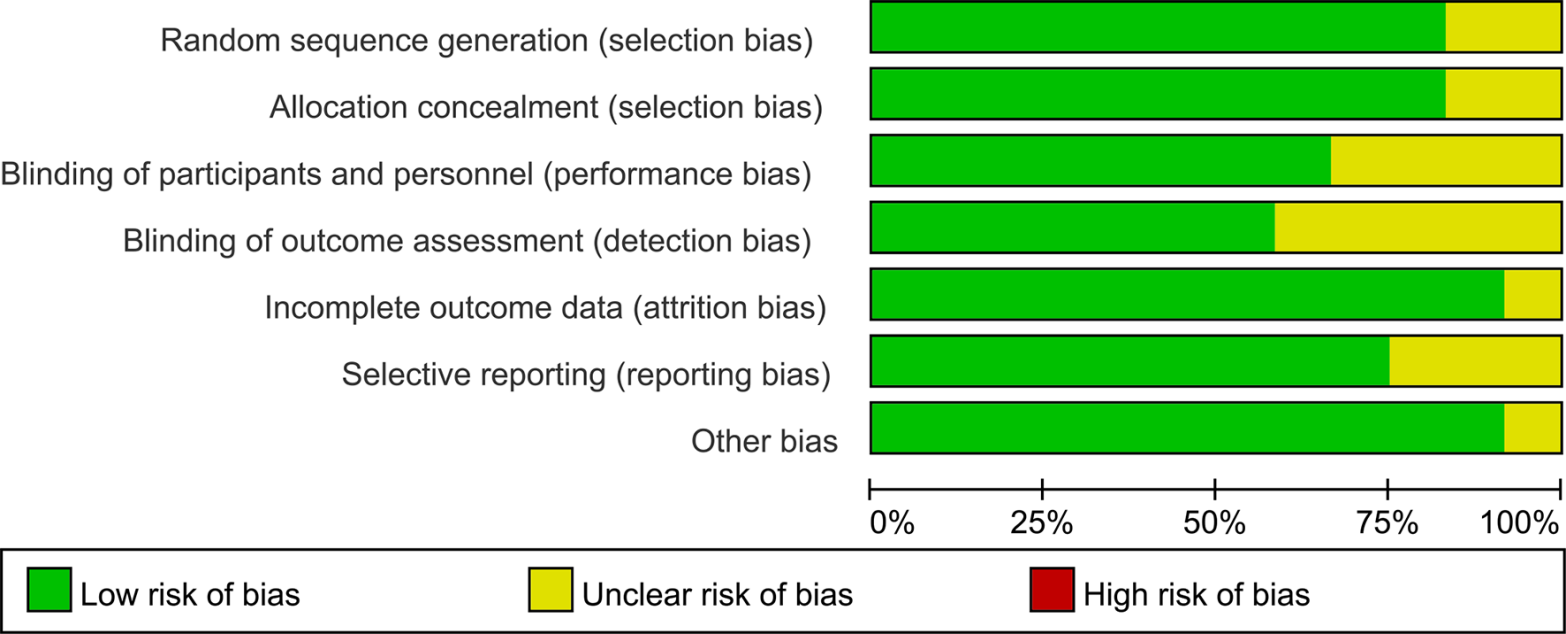
Risk of bias assessment across included studies.

Assessment of risk of bias is summarized in **Figure 2**. Method of random sequence generation was reported and found to be adequate in 12 studies. Ten trials provided sufficient information concerning the proceedings of allocation concealment. Eight studies blinded study participants and personnel to the dietary interventions. Seven studies reported blinding of outcome assessors. One study had an unclear risk of attrition bias as the result of incomplete reporting of outcome data, as the dropout rate placebo group is higher. Selective reporting was found in ten trials.

### Meta-Analysis

**Figure 3:**
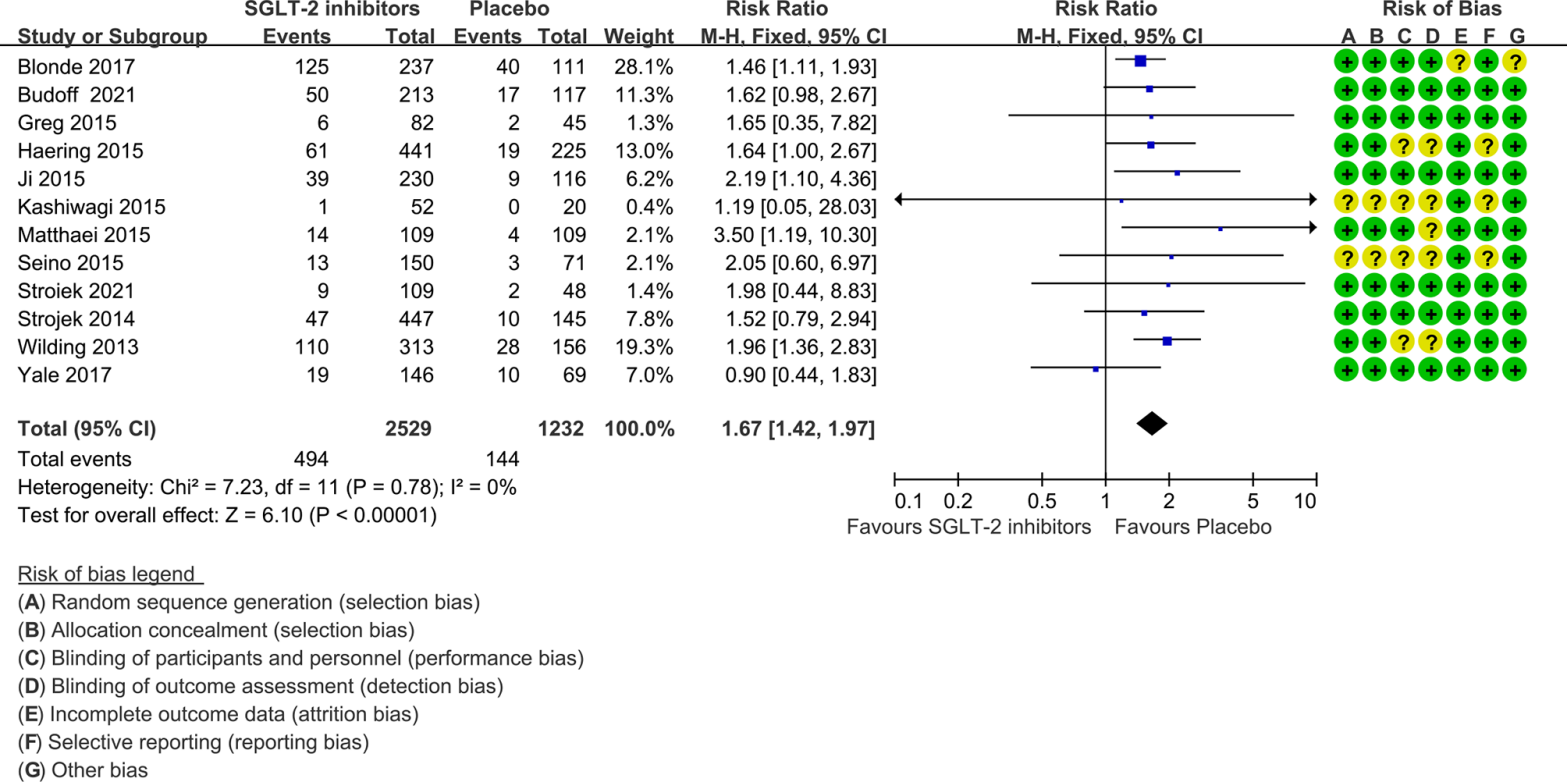
Risk of hypoglycemia in all included trials. Forest plot showing risk of hypoglycemia in patients treated with SGLT-2 inhibitors plus sulfonylureas compared with placebo plus sulfonylureas.

The risk ratio of hypoglycemia for SGLT-2 inhibitors plus sulfonylureas versus placebo plus sulfonylureas was 1.67 (1.42 to 1.97), with no evidence of heterogeneity across the trials (P=0.78, I^2 =^ 0%; **Figure 3**).

The NNH was 13 (95% confidence interval 9 to 21) for a follow-up period of 24 weeks or less, 11 (8 to 18) for 25 to 48 weeks, and 7 (5 to 10) for more than 48 weeks.

**Figure 4:**
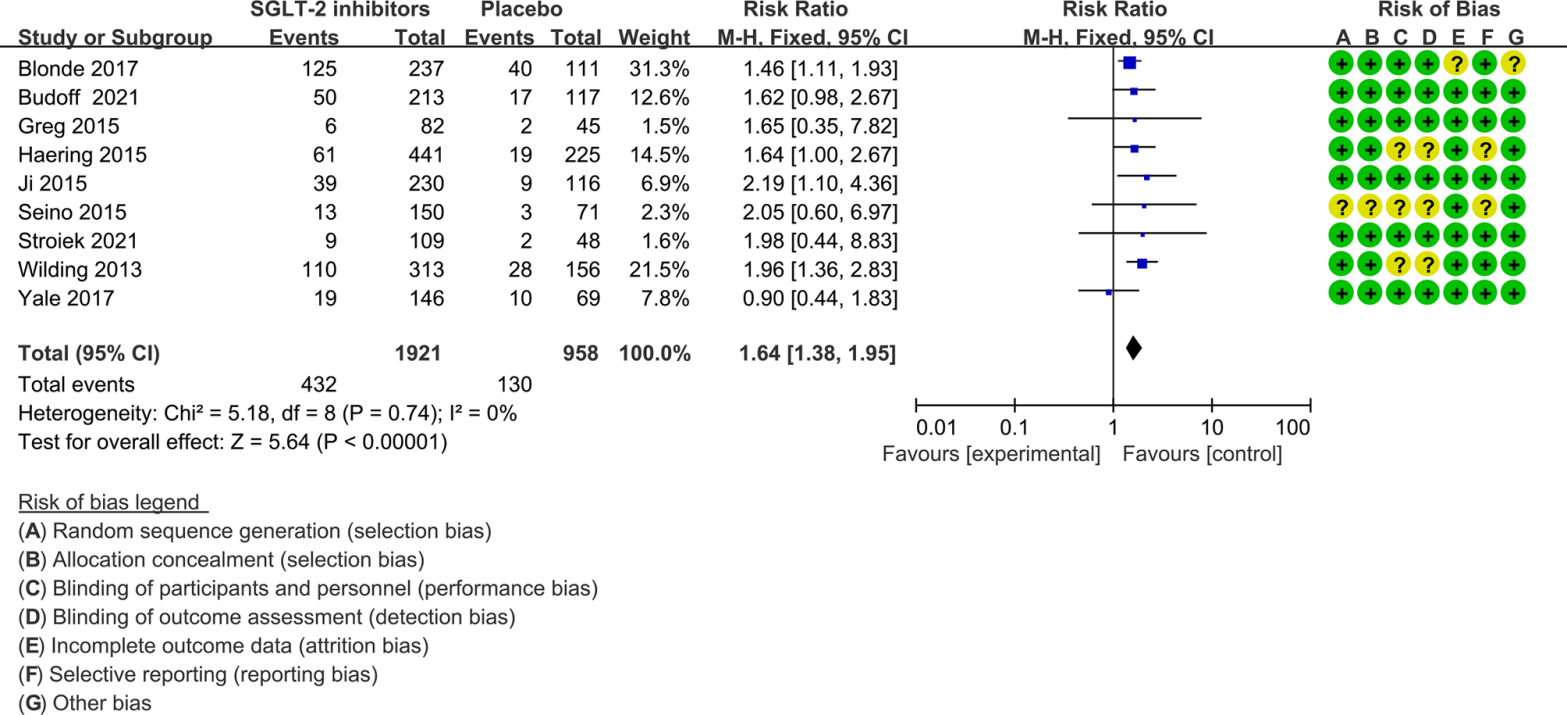
Risk of hypoglycemia in trials with the same definition of hyperglycemia. Forest plot showing risk of hypoglycemia in patients treated with SGLT-2 inhibitors plus sulfonylureas compared with placebo plus sulfonylureas (Trials with different or not report a definition of hypoglycemia were excluded).

**Figure 5:**
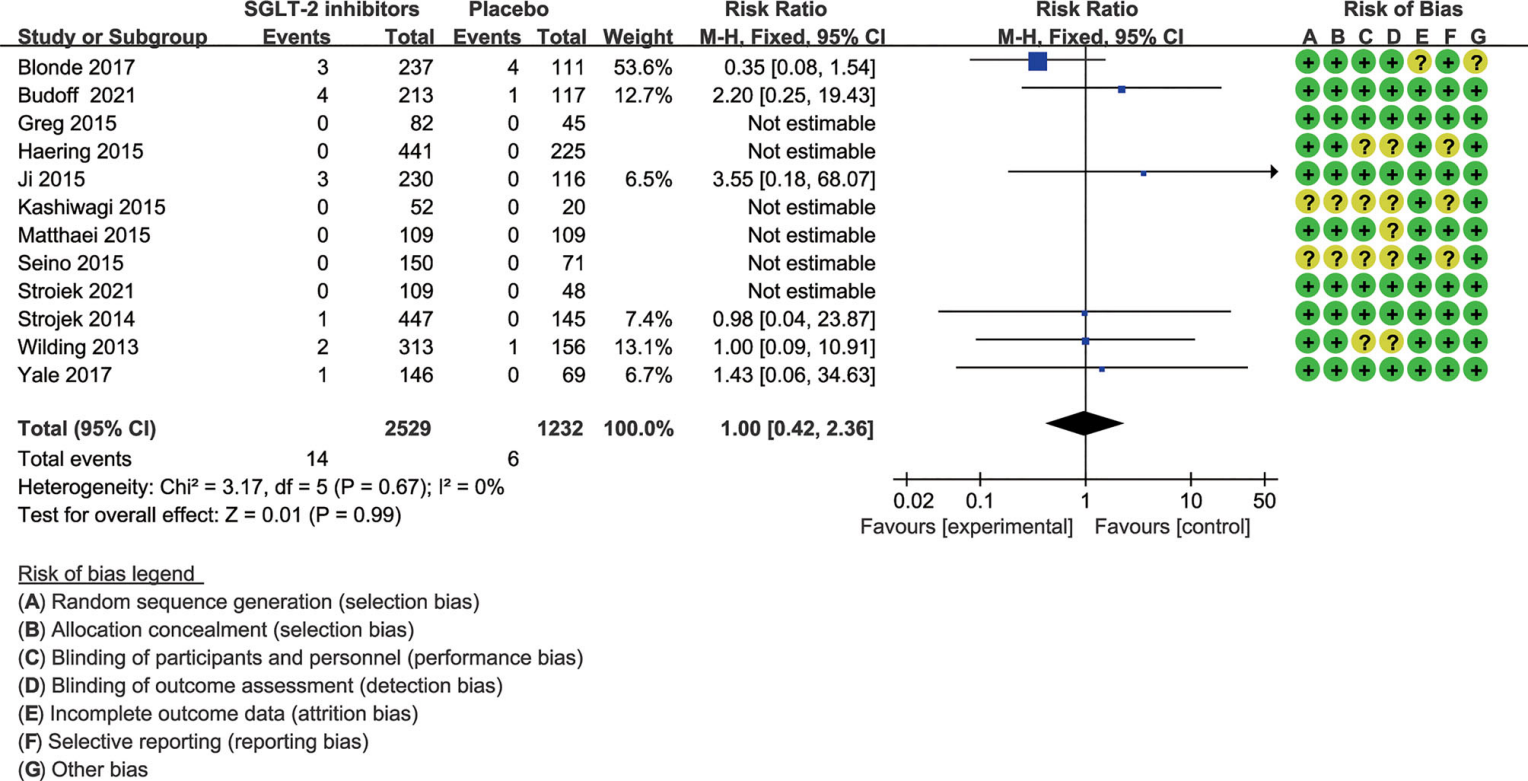
Risk of severe hypoglycemia. Forest plot showing risk of severe hypoglycemia in patients treated with SGLT-2 inhibitors plus sulfonylureas compared with placebo plus sulfonylureas.

In nine of the 12 studies definitions of hypoglycemia (≤ 3.9 mmol/l with or without symptoms) are the same, while the remaining three studies have different or do not report a definition of hypoglycemia. The risk ratio was 1.64(1.38 to 1.95) when trials with different or not report a definition of hypoglycemia were excluded (**Figure 4**). Severe hypoglycemic events were reported in six of the 12 studies (**Table 2**). In addition, the risk of severe hypoglycemia did not have significant difference between the two groups (**Figure 5**).

**Figure 6:**
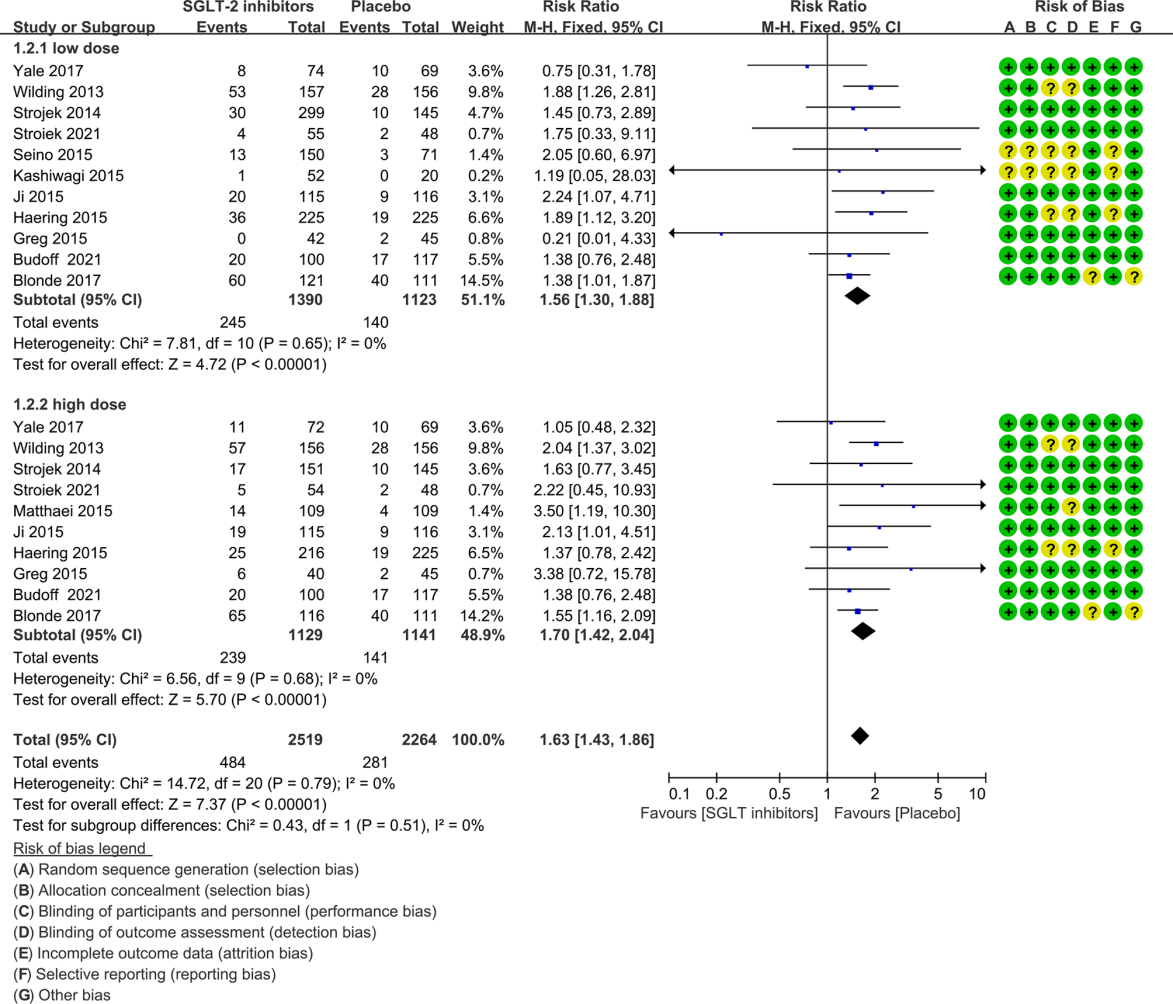
Risk of hypoglycemia in subgroups. Forest plot showing risk of hypoglycemia in patients treated with higher or lower SGLT-2 inhibitors plus sulfonylureas compared with placebo plus sulfonylureas.

In subgroup analysis, there was no difference between lower and higher dose SGLT-2 inhibitors for risk of hypoglycemia (P=0.51, I^2 =^ 0%; **Figure 6**). The risk for SGLT-2 inhibitors remained statistically significantly increased at lower dose (1.56, 1.30 to 1.88) and higher dose (1.70, 1.43 to 1.86).

**Figure 7:**
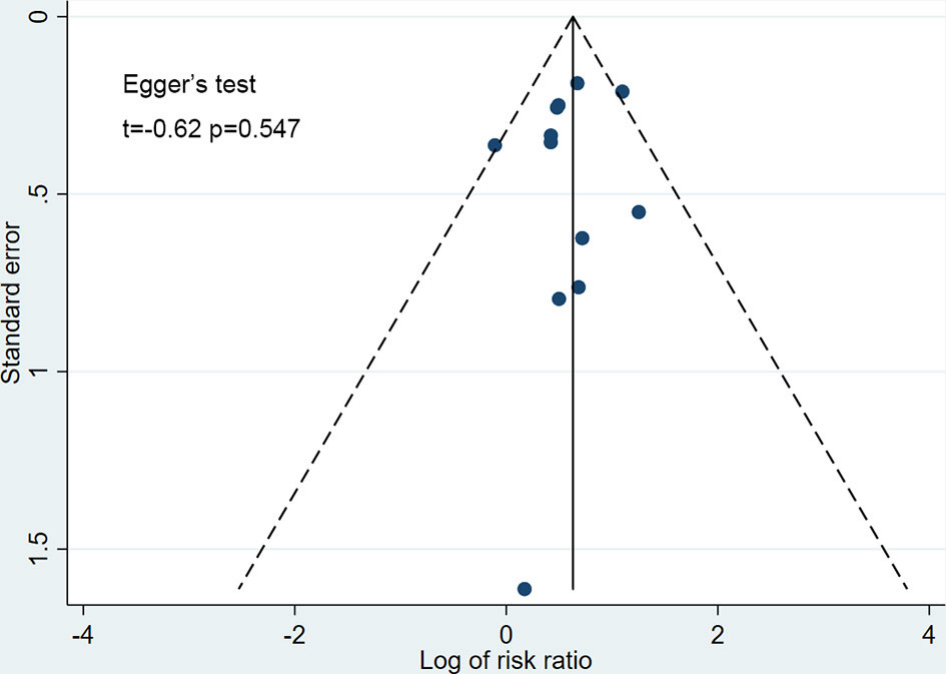
Funnel plot for publication bias. Scatter plot reporting risk ratio of the studies testing SGLT-2 inhibitors plus sulfonylureas compared with placebo plus sulfonylureas (horizontal axis) against their standard error (vertical axis).

The funnel plots for all studies did not show any asymmetry (**Figure 7**), and the Egger’s test did not suggest any publication bias (t=-0.62, p=0.547). The strength of evidence of this meta-analysis was graded as high according to GRADE (**eTable 2** in the supplement).

## Discussion

This systemic review and meta-analysis shows that over a 50% increase in the risk of hypoglycemia when an SGLT-2 inhibitor was added to sulfonylureas to treat diabetic patients, and this combination therapy lead to one more case of hypoglycemia for every 13 patients for treatment duration less than first six months of treatment. The present study is based on a large number of patients, which enables us to arrive plausible conclusions. Other strengths included the high quality of each study, which was assessed according to the Cochrane Collaboration tool for risk of bias assessment. In addition, funnel plots for the different outcomes did not suggest any publication bias. In conclusion, the quality of evidence of the present study was rated as high according to GRADE.

A number of limitations do exist in the present meta-analysis. First, because we cannot get the data for the risk of hypoglycemia in patients receiving sulfonylureas, five studies could not be included (36–40). However, given the size of the present meta-analysis and the high number of hypoglycemia cases, including results from these studies may not be able to change the results. There was no heterogeneity across trials further, which supports this hypothesis. Second, the definition of hypoglycemia varied among the included trials, and it was not reported in one. However, an analysis excluded these trials did not substantially change the results of the meta-analysis (**Figure 4**). Third, the results might be more strongly affected by trials with lager participants, although our sensitivity analysis found that removing any trial did not substantially change the results of the meta-analysis. Fourth, the incidence of hypoglycemia is different among studies; however, this did not affect the estimation of the pooled risk nor on the NNH, which was calculated according to an assumed control risk of hypoglycemia from a meta-analysis with 27 clinical trials included (34). Fifth, the effects of different sulfonylureas and different levels of renal function on the study results cannot be determined because of a lack of sufficient data.

SGLT-2 inhibitors increase the glucose excretion to lower hyperglycemia, which is a unique insulin-independent mode of action (41) with a limited risk of hyperglycemia. Because of the beneficial effects on cardio-renal outcomes, the guidelines propose a new paradigm in diabetes management with a preferential place for SGLT-2 inhibitors. However, insulin secretion is already stimulated independently of glycemia in patients treated with sulfonylureas, and the addition of a glucose execration effect may result in an increase in the incidence of hypoglycemia. Given the extensive use of sulfonylureas and preferential place of SGLT-2 inhibitors for the treatment of type 2 diabetes, the risk associated with the combination therapy would result in a substantial number of cases of induced hypoglycemia.

Addition of an SGLT-2 inhibitor to sulfonylureas increase the risk of hypoglycemia, which is described in the product characteristics for SGLT-2 inhibitors. From our study, we knew that the low doses of SGLT-2 inhibitors also increased hypoglycemia risk statistically significant. Therefore, we may recommend using full dose SGLT-2 inhibitors but a reduced sulfonylurea dose in patients taking such combinations. In consistent with our findings, a recent study suggested that maintaining sulfonylurea treatment at the lowest dose could ensure better glycemic control without increasing hypoglycemia in patients treated with such combinations (42). However, owing to the small number of cases and other limitations, further studies are required to confirm the conclusions.

Hyperglycemia increases the risk of hospital admissions and cardiovascular mortality. In addition, it is able to activate the renin-angiotensin-aldosterone and sympathetic systems, which leads to higher risk of cardiovascular events. The risk of severe hypoglycemia does not increase significantly in the present study. However, mild or moderate hypoglycemia is able to decrease the usual adrenergic response to hyperglycemia. It may induce hypoglycemia unawareness and compromise behavioral responses, which can result in severe hypoglycemia. It is inadvisable to achieve optimal glycemic control at the expense of hypoglycemic events. Overall, the clinicians should pay attention to the information of any degree of hypoglycemia.

## Conclusion

In conclusion, the addition of SGLT-2 inhibitors to sulfonylureas was associated with an increased risk of hypoglycemia in type 2 diabetic patients. In addition, the combination therapy would lead to one more case of hypoglycemia in every 13 treated patients in the first 24 weeks of treatment. This negative effect may induce a remarkedly increase the cases of hypoglycemia worldwide. These outcomes strongly suggest that dose reduction of sulfonylureas is important when adding an SGLT-2 inhibitor to sulfonylurea treatment, and it is important to determine the efficacy of this measure in minimizing the risk of hypoglycemia.

## Data Availability Statement

The raw data supporting the conclusions of this article will be made available by the authors, without undue reservation.

## Author Contributions

ML and MJ had full access to all the data in the study and takes responsibility for the integrity of the data and the accuracy of the data analysis. MJ and ML conceived the research idea. All authors performed the meta-analysis and the draft manuscript. All authors contributed to the article and approved the submitted version.

## Funding

This study was supported by grant 2017SK1020 from Department of Science and Technology of Hunan province.

## Conflict of Interest

The authors declare that the research was conducted in the absence of any commercial or financial relationships that could be construed as a potential conflict of interest.

## Publisher’s Note

All claims expressed in this article are solely those of the authors and do not necessarily represent those of their affiliated organizations, or those of the publisher, the editors and the reviewers. Any product that may be evaluated in this article, or claim that may be made by its manufacturer, is not guaranteed or endorsed by the publisher.
