# Supplementary material for: Adding Sodium–Glucose Co-Transporter 2 Inhibitors to Sulfonylureas and Risk of Hypoglycemia: A Systematic Review and Meta-Analysis of Randomized Controlled Trials
Source: Front Endocrinol (Lausanne). 2021 Oct 21;12:713192. doi: 10.3389/fendo.2021.713192 (PMC8568344; doi:10.3389/fendo.2021.713192)
Supplement: Supplementary file 1 [file DataSheet_1.pdf]

eFigure 1: Search Strategy

PubMed

#1 sglt[Title/Abstract]
#2 sglt2[Title/Abstract]
#3 Sodium-Glucose Transporter 2[Title/Abstract]
#4 Sodium Glucose Cotransporters[Title/Abstract]
#5 Sodium Glucose Transporters[Title/Abstract]
#6 Sodium Glucose Transport System[Title/Abstract]
#7 SGLT Proteins[Title/Abstract]
#8 Glucose-Sodium Transport System[Title/Abstract]
#9 "Sodium Glucose Transport Proteins*"[Title/Abstract]
#10 Sodium-Glucose Transport Proteins [MeSH Terms]
#11 Ipragliflozin[Title/Abstract]
#12 Suglat[Title/Abstract])
#13 ASP1941[Title/Abstract]
#14 ASP-1941[Title/Abstract]
#15 empagliflozin[Title/Abstract]
#16 BI10773[Title/Abstract]
#17 BI-10773[Title/Abstract])
#18 Jardiance[Title/Abstract]
#19 Phlorhizin[Title/Abstract]
#20 Phloridzin [Title/Abstract]
#21 tofogliflozin[Title/Abstract]
#22 CSG452[Title/Abstract]
#23 CSG-452[Title/Abstract]
#24 Apleway[Title/Abstract]
#25 Deberza[Title/Abstract]
#26 R-7201[Title/Abstract]
#27 R7201[Title/Abstract])))
#28 sergliflozin[Title/Abstract]
#29 GW869682[Title/Abstract]
#30 GW-869682[Title/Abstract]
#31 KGT1251[Title/Abstract]
#32 KGT-1251[Title/Abstract]
#33 ertugliflozin[Title/Abstract]
#34 PF-04971729[Title/Abstract]
#35 PF04971729[Title/Abstract]
#36 MK-8835[Title/Abstract]
#37 MK8835[Title/Abstract]
#38 WAY-123783[Title/Abstract]
#39 WAY123783[Title/Abstract]
#40 TS-071[Title/Abstract]

#41 TS071[Title/Abstract]
#42 sotagliflozin[Title/Abstract])
#43 LX-4211[Title/Abstract]
#44 LX4211[Title/Abstract]
#45 LP-802034[Title/Abstract]
#46 LP802034[Title/Abstract]
#47 T-1095[Title/Abstract]
#48 T1095[Title/Abstract]
#49 ISIS-SGLT2Rx[Title/Abstract]
#50 ISISSGLT2Rx[Title/Abstract]
#51 ISIS 388626[Title/Abstract]
#52 ISIS-388626[Title/Abstract]
#53 YM543[Title/Abstract]
#54 YM-543[Title/Abstract]
#55 AVE2268[Title/Abstract]
#56 AVE-2268[Title/Abstract]
#57 dapagliflozin[Title/Abstract]
#58 BMS512148[Title/Abstract]
#59 BMS-512148[Title/Abstract]
#60 Canagliflozin[Title/Abstract]
#61 Invokana[Title/Abstract]
#62 JNJ-28431754[Title/Abstract]
#63 TA-7284[Title/Abstract]
#64 JNJ 28431754[Title/Abstract]
#65 TA7284[Title/Abstract]
#66 luseogliflozin[Title/Abstract]
#67 Lusefi[Title/Abstract])
#68 TS-071[Title/Abstract]
#69 TS071[Title/Abstract]
#70 bexagliflozin[Title/Abstract]
#71 EGT0001442[Title/Abstract]
#72 EGT-0001442[Title/Abstract]
#73 THR-1442[Title/Abstract]
#74 THR1442[Title/Abstract]
#75 EGT0001474[Title/Abstract]
#76 EGT-0001474[Title/Abstract]
#77 OR/#1-76
#78 "randomized controlled trial"[Publication Type]
#79 "randomized controlled trials as topic"[MeSH Terms]
#80 "randomised clinical trials"[All Fields]
#81 "randomized clinical trials"[All Fields])
#82 OR/78-81
#83 #75 AND #80

EMBASE

#1	**'sglt':ab,ti**
#2	**'sglt2':ab,ti**
#3	**'sodium-glucose transporter 2':ab,ti**
#4	**'sodium glucose cotransporters':ab,ti**
#5	**'sodium glucose transporters':ab,ti**
#6	**'sodium glucose transport system':ab,ti**
#7	**'sglt proteins':ab,ti**
#8	**'glucose-sodium transport system':ab,ti**
#9	**'sodium glucose transport proteins*':ab,ti**
#10	**'sodium-glucose transport proteins*':ab,ti**
#11	**'ipragliflozin':ab,ti**
#12	**'suglat':ab,ti**
#13	**'asp1941':ab,ti**
#14	**'asp-1941':ab,ti**
#15	**'empagliflozin':ab,ti**
#16	**'bi10773':ab,ti**
#17	**'bi-10773':ab,ti**
#18	**'jardiance':ab,ti**
#19	**'phlorhizin':ab,ti**
#20	**'phloridzin':ab,ti**
#21	**'tofogliflozin':ab,ti**
#22	**'csg452':ab,ti**
#23	**'csg-452':ab,ti**
#24	**'apleway':ab,ti**
#25	**'deberza':ab,ti**
#26	**'r-7201':ab,ti**
#27	**'r7201':ab,ti**
#28	**'sergliflozin':ab,ti**
#29	**'gw869682':ab,ti**
#30	**'gw-869682':ab,ti**
#31	**'kgt1251':ab,ti**
#32	**'kgt-1251':ab,ti**
#33	**'ertugliflozin':ab,ti**
#34	**'pf-04971729':ab,ti**
#35	**'pf04971729':ab,ti**
#36	**'mk-8835':ab,ti**
#37	**'mk8835':ab,ti**
#38	**'way-123783':ab,ti**
#39	**'way123783':ab,ti**
#40	**'ts-071':ab,ti**
#41	**'ts071':ab,ti**
#42	**'sotagliflozin':ab,ti**

**#43**	**'lx-4211':ab,ti**
**#44**	**'lx4211':ab,ti**
**#45**	**'lp-802034':ab,ti**
**#46**	**'lp802034':ab,ti**
**#47**	**'t-1095':ab,ti**
**#48**	**'t1095':ab,ti**
**#49**	**'isis-sglt2rx':ab,ti**
**#50**	**'isissglt2rx':ab,ti**
**#51**	**'isis388626':ab,ti**
**#52**	**'isis-388626':ab,ti**
**#53**	**'ym-543':ab,ti**
**#54**	**'ym543':ab,ti**
**#55**	**'ave-2268':ab,ti**
**#56**	**'ave2268':ab,ti**
**#57**	**'dapagliflozin':ab,ti**
**#58**	**'bms512148':ab,ti**
**#59**	**'bms-512148':ab,ti**
**#60**	**'canagliflozin':ab,ti**
**#61**	**'invokana':ab,ti**
**#62**	**'jnj-28431754':ab,ti**
**#63**	**'jnj28431754':ab,ti**
**#64**	**'ta-7284':ab,ti**
**#65**	**'ta7284':ab,ti**
**#66**	**'luseogliflozin':ab,ti**
**#67**	**'lusefi':ab,ti**
**#68**	**'ts-071':ab,ti**
**#69**	**'ts071':ab,ti**
**#70**	**'bexagliflozin':ab,ti**
**#71**	**'egt0001442':ab,ti**
**#72**	**'egt-0001442':ab,ti**
**#73**	**'thr-1442':ab,ti**
**#74**	**'thr1442':ab,ti**
**#75**	**'egt0001474':ab,ti**
**#76**	**'egt-0001474':ab,ti**
**#77**	**#1 OR #2 - #76**
**#78**	**'randomized controlled trial'/exp**
**#79**	**#77 AND #78**

The Cochrane library

**#1**	**MeSH descriptor: [Sodium-Glucose Transport Proteins] explode all trees**
**#2**	**sglt:ti,ab,kw (Word variations have been searched)**
**#3**	**sglt 2:ti,ab,kw (Word variations have been searched)**
**#4**	**Sodium Glucose Transport Protein*:ti,ab,kw (Word variations have been searched)**
**#5**	**Glucose-Sodium Transport System:ti,ab,kw (Word variations have been searched)**
**#6**	**SGLT Proteins:ti,ab,kw (Word variations have been searched)**
**#7**	**Sodium Glucose Transporters:ti,ab,kw (Word variations have been searched)**
**#8**	**Sodium Glucose Cotransporters:ti,ab,kw (Word variations have been searched)**
**#9**	**Sodium-Glucose Transporter 2:ti,ab,kw (Word variations have been searched)**
**#10**	**dapagliflozin*:ti,ab,kw (Word variations have been searched)**
**#11**	**BMS 512148:ti,ab,kw (Word variations have been searched)**
**#12**	**BMS512148:ti,ab,kw (Word variations have been searched)**
**#13**	**canagliflozin*:ti,ab,kw (Word variations have been searched)**
**#14**	**JNJ-28431754:ti,ab,kw (Word variations have been searched)**
**#15**	**TA-7284:ti,ab,kw (Word variations have been searched)**
**#16**	**JNJ28431754:ti,ab,kw (Word variations have been searched)**
**#17**	**TA7284:ti,ab,kw (Word variations have been searched)**
**#18**	**Ipragliflozin*:ti,ab,kw (Word variations have been searched)**
**#19**	**ASP1941:ti,ab,kw (Word variations have been searched)**
**#20**	**ASP-1941:ti,ab,kw (Word variations have been searched)**
**#21**	**empagliflozin*:ti,ab,kw (Word variations have been searched)**
**#22**	**bi10773:ti,ab,kw (Word variations have been searched)**
**#23**	**BI-10773:ti,ab,kw (Word variations have been searched)**
**#24**	**Jardiance*:ti,ab,kw (Word variations have been searched)**
**#25**	**bexagliflozin*:ti,ab,kw (Word variations have been searched)**
**#26**	**EGT0001442:ti,ab,kw (Word variations have been searched)**
**#27**	**EGT-0001442:ti,ab,kw (Word variations have been searched)**
**#28**	**THR-1442:ti,ab,kw (Word variations have been searched)**
**#29**	**THR1442:ti,ab,kw (Word variations have been searched)**
**#30**	**EGT0001474:ti,ab,kw (Word variations have been searched)**
**#31**	**EGT-0001474:ti,ab,kw (Word variations have been searched)**
**#32**	**WAY-123783:ti,ab,kw (Word variations have been searched)**
**#33**	**WAY123783:ti,ab,kw (Word variations have been searched)**
**#34**	**TS-071:ti,ab,kw (Word variations have been searched)**
**#35**	**TS071:ti,ab,kw (Word variations have been searched)**
**#36**	**Phlorhizin*:ti,ab,kw (Word variations have been searched)**

**#37**	**Phloridzin*:ti,ab,kw (Word variations have been searched)**
**#38**	**tofogliflozin*:ti,ab,kw (Word variations have been searched)**
**#39**	**CSG452:ti,ab,kw (Word variations have been searched)**
**#40**	**CSG-452:ti,ab,kw (Word variations have been searched)**
**#41**	**Apleway*:ti,ab,kw (Word variations have been searched)**
**#42**	**Deberza*:ti,ab,kw (Word variations have been searched)**
**#43**	**R-7201:ti,ab,kw (Word variations have been searched)**
**#44**	**R 7201:ti,ab,kw (Word variations have been searched)**
**#45**	**sotagliflozin*:ti,ab,kw (Word variations have been searched)**
**#46**	**LX-4211:ti,ab,kw (Word variations have been searched)**
**#47**	**LX4211:ti,ab,kw (Word variations have been searched)**
**#48**	**LP802034:ti,ab,kw (Word variations have been searched)**
**#49**	**T-1095:ti,ab,kw (Word variations have been searched)**
**#50**	**LP-802034:ti,ab,kw (Word variations have been searched)**
**#51**	**T1095:ti,ab,kw (Word variations have been searched)**
**#52**	**ISIS-SGLT2Rx:ti,ab,kw (Word variations have been searched)**
**#53**	**ISISSGLT2Rx:ti,ab,kw (Word variations have been searched)**
**#54**	**ISIS 388626:ti,ab,kw (Word variations have been searched)**
**#55**	**ISIS-388626:ti,ab,kw (Word variations have been searched)**
**#56**	**ertugliflozin*:ti,ab,kw (Word variations have been searched)**
**#57**	**PF-04971729:ti,ab,kw (Word variations have been searched)**
**#58**	**PF04971729:ti,ab,kw (Word variations have been searched)**
**#59**	**MK-8835:ti,ab,kw (Word variations have been searched)**
**#60**	**MK8835:ti,ab,kw (Word variations have been searched)**
**#61**	**YM543:ti,ab,kw (Word variations have been searched)**
**#62**	**YM-543:ti,ab,kw (Word variations have been searched)**
**#63**	**AVE2268:ti,ab,kw (Word variations have been searched)**
**#64**	**remogliflozin*:ti,ab,kw (Word variations have been searched)**
**#65**	**GSK 189075A:ti,ab,kw (Word variations have been searched)**
**#66**	**GSK-189075A:ti,ab,kw (Word variations have been searched)**
**#67**	**BHV-091009:ti,ab,kw (Word variations have been searched)**
**#68**	**BHV 091009:ti,ab,kw (Word variations have been searched)**
**#69**	**KGT-1681:ti,ab,kw (Word variations have been searched)**
**#70**	**KGT1681:ti,ab,kw (Word variations have been searched)**
**#71**	**sergliflozin:ti,ab,kw (Word variations have been searched)**
**#72**	**GW869682:ti,ab,kw (Word variations have been searched)**
**#73**	**GW-869682:ti,ab,kw (Word variations have been searched)**
**#74**	**KGT1251:ti,ab,kw (Word variations have been searched)**
**#75**	**KGT-1251:ti,ab,kw (Word variations have been searched)**
**#76**	**luseogliflozin*:ti,ab,kw (Word variations have been searched)**
**#77**	**Lusefi*:ti,ab,kw (Word variations have been searched)**
**#78**	**#1 or #2 - #77**

Clinicaltrial. gov

"tofogliflozin" OR "empagliflozin" OR "dapagliflozin" OR "canagliflozin" OR "sotagliflozin" OR "luseogliflozin" OR "Ipragliflozin" OR "Sergliflozin" OR "ertugliflozin"
