# Supplementary material for: Adding Sodium–Glucose Co-Transporter 2 Inhibitors to Sulfonylureas and Risk of Hypoglycemia: A Systematic Review and Meta-Analysis of Randomized Controlled Trials
Source: Front Endocrinol (Lausanne). 2021 Oct 21;12:713192. doi: 10.3389/fendo.2021.713192 (PMC8568344; doi:10.3389/fendo.2021.713192)
Supplement: Supplementary file 2 [file Table_1.docx]

| eTable 1. lower and higher dose of SGLT-2 inhibitors | | |
| --- | --- | --- |
|  | Low dose (mg/d) | Full dose (mg/d) |
| luseogliflozin | 2.5 | 5 |
| dapagliflozin | 2.5 and 5 | 10 |
| ipragliflozin | 50 | 100 |
| canagliﬂozin | 100 | 300 |
| empagliflozin | 10 | 25 |
| Ertugliflozin | 5 | 15 |
