# Supplementary material for: Adding Sodium–Glucose Co-Transporter 2 Inhibitors to Sulfonylureas and Risk of Hypoglycemia: A Systematic Review and Meta-Analysis of Randomized Controlled Trials
Source: Front Endocrinol (Lausanne). 2021 Oct 21;12:713192. doi: 10.3389/fendo.2021.713192 (PMC8568344; doi:10.3389/fendo.2021.713192)
Supplement: Supplementary file 3 [file Table_2.docx]

| **eTable 2. Summary of findings in 12 randomized controlled trials of hypoglycemia according to GRADE framework** | | | | | | | | | | | | | |
| --- | --- | --- | --- | --- | --- | --- | --- | --- | --- | --- | --- | --- | --- |
| **Quality assessment** | | | | | **No of patients** | | **Effect** | |  |  | |  |  |
| **Risk of bias** | **Inconsistency** | **Indirectness** | **Imprecision** | **Other considerations** | **Risk of hypoglycemia** | **Placebo** | **Relative (95% CI)** | **Absolute**  **(95% CI)** | **Quality** | |  | **Importance** |  |
| no serious* | no serious† | no serious | no serious‡ | none | 494/2529  (19.5%) | 144/1232  (11.7%) | 1.67 (1.4 to 1.97) | 78 more per 1000 (from 49 more to 113 more) | High | | | Critical§ | |

*No study was judged to have a high risk of detection bias. †No heterogeneity was found among studies. ‡Sample size is large (n=3761), number of events high (n=638), and confidence intervals of pooled risk ratio clearly do not cross the line of no effect (lower bound of 95% confidence interval 1.42) §Hypoglycemia is the most common adverse reaction related to glucose lowering treatment. It increases the risk of all-cause mortality and cardiovascular events. Symptoms related to hypoglycemia (eg, nervousness, sweating, trembling, weakness, palpitations) affect quality of life.
